# No evidence that visual impulses enhance the readout of retrieved long-term memory contents from EEG activity

**DOI:** 10.1162/imag_a_00330

**Published:** 2024-10-24

**Authors:** Sander van Bree, Abbie Sarah Mackenzie, Maria Wimber

**Affiliations:** Centre for Cognitive Neuroimaging, School of Psychology and Neuroscience, University of Glasgow, Glasgow, United Kingdom; Centre for Human Brain Health, School of Psychology, Birmingham, United Kingdom; Department of Medicine, Justus Liebig University, Giessen, Germany; Max Planck Institute for Human Cognitive and Brain Sciences, Leipzig, Germany

**Keywords:** long-term memory, MVPA, decoding, EEG, ping, visual impulse, perturbation, brain dynamics

## Abstract

The application of multivariate pattern analysis (MVPA) to electroencephalography (EEG) data allows neuroscientists to track neural representations at temporally fine-grained scales. This approach has been leveraged to study the locus and evolution of long-term memory contents in the brain, but a limiting factor is that decoding performance remains low. A key reason for this is that processes such as encoding and retrieval are intrinsically dynamic across trials and participants, and this runs in tension with MVPA and other techniques that rely on consistently unfolding neural codes to generate predictions about memory contents. The presentation of visually perturbing stimuli may experimentally regularize brain dynamics, making neural codes more stable across measurements to enhance representational readouts. Such enhancements, which have repeatedly been demonstrated in working memory contexts, could offer a tool to improve decoding in long-term memory tasks. In this study, we evaluated whether visual perturbations—or*pings*—improve our ability to predict the category of retrieved images from EEG activity during cued recall. Overall, our findings suggest that while pings evoked a prominent neural response, they did not reliably produce improvements in MVPA-based classification across several analyses. We discuss possibilities that could explain these results, including the role of experimental and analysis parameter choices and mechanistic differences between working and long-term memory.

## 1 Introduction

A central question in memory research is how the brain retrieves information stored in long-term memory (LTM) in the service of adaptive behaviour. This research topic has inspired work from a variety of angles, involving different experimental protocols and methods—including neuroimaging modalities. Electroencephalography (EEG) and magnetoencephalography (MEG) have proven an integral part of this project because they capture brain dynamics on a sub-second resolution. Such granularity is crucial, given that memory retrieval typically unfolds on the order of seconds, with the neural cascades underpinning memory retrieval evolving even faster (Staresina & Wimber, 2019).

To study the evolution of retrieved contents in the brain, one widely pursued family of techniques is multivariate pattern analysis (MVPA)—more broadly known as classification or decoding (Grootswagers et al., 2017;Haxby et al., 2014). These tools extract and upweight signal dimensions that robustly covary with retrieved memory contents, effectively boosting the signal-to-noise ratio of associated neural activity. MVPA has been successfully used to enrich our understanding of memory, including how information is encoded (Fritch et al., 2020;Kragel et al., 2017;Kuhl et al., 2012), consolidated (Deuker et al., 2013;Maguire, 2014;Schreiner et al., 2021), and reinstated during memory recall (i.e., pattern completion;Danker & Anderson, 2010;Favila et al., 2020;Rissman & Wagner, 2012;Xue, 2018).

Despite such advancements, the decoding of long-term memory contents in electrophysiology data typically remains only slightly above chance, impairing our ability to study the evolution of neural patterns of interest. One reason for this limitation is that memory processes and their associated brain activity are highly dynamic, which results in variable patterns across trials and participants (Madore & Wagner, 2022;Ter Wal et al., 2021). Indeed, MVPA and most other EEG-based analyses rely for their robust predictions on the existence of a detectably constant cascade of neural patterns across measurements (van Bree et al., 2022). This clash between variability in neural processes on the one hand and the constancy assumption of our analyses on the other may cause us to miss representations of interest, or to obtain different results depending on what experimental event we timelock EEG data to (e.g., retrieval cues vs. button presses;Linde-Domingo et al., 2019). A factor that further hampers our ability to robustly decode representations is that retrieval comes with fainter neural patterns to begin with compared with perception (Favila et al., 2020,^2022^;Pearson et al., 2015). Together, these points invite creative techniques that improve our ability to infer long-term memory representations from dynamic brain activity.

In this study, we explore a perturbational method that has the potential to mitigate two issues at the same time: low signal fidelity at the level of measurement and variability in neural processing dynamics. Specifically, in this EEG study, we evaluated whether the presentation of a high contrast visual stimulus—henceforth referred to as a “ping”—during LTM retrieval enhances the readout of signatures of retrieved content. In motivating the hypothesis that*pings*boost the decodability of LTM representations, we built directly onto recent successful efforts in the domain of working memory (WM). In that context, pings have been used to enhance the decodability of the orientation (Ten Oever et al., 2020;Wolff et al., 2015,^2017^,^2020^;Yang et al., 2023) and colour (Kandemir et al., 2023) of objects actively maintained in WM, as well as anticipated target locations (Duncan et al., 2023). A preliminary explanation for these findings is that pings induce a robust evoked response that interacts and indeed boosts the footprint of active neural representations, enhancing their SNR (Barbosa et al., 2021). Specifically, pings may regularize neural dynamics across trials and participants by producing a phase reset of brain oscillations that coordinate information processing across neuronal populations. In support of this, visual stimuli presented during memory tasks have been shown to reset the phase of low-frequency brain oscillations that are implicated in encoding and retrieval (Haque et al., 2015;Rizzuto et al., 2003; audiovisual stimuli inCruzat et al., 2021). Thus, by inducing pings at experimentally controlled moments, researchers may gain a level of control over variability in synchronized activity across information-coding neurons, making their dynamics more similar across measurements to improve the predictive power of MVPA.

While ping-based methods have been shown to work in WM contexts, a crucial question is whether the technique generalizes to LTM research in which information is retrieved from stored representations. In a previous study, it was shown that pings can improve the decoding of grating orientations from LTM when these contents were crossmodally reactivated in WM through a paired auditory stimulus (Kandemir & Akyürek, 2023). In the present work, we systematically explored further whether pings can enhance the classification of re-activated LTM traces by (i) contrasting trials with and without pings, (ii) applying the pings at variable retrieval times, (iii) using a memory task that involves more semantic richness, and (iv) by varying a large array of analysis and decoding parameters. On the whole, we find no compelling evidence that pings boost the classification of retrieved image pairs from EEG activity.

## 2 Methods

### 2.1 Participants

We recruited 33 volunteers (22 women, M_age_= 23.8 years, SD_age_= 2.6 years, range = 18 to 31) with normal or corrected-to-normal vision, and with no history of epileptic attacks or neuropsychological conditions that could interfere with the examined study effects. The sample size required to derive a reliable effect was estimated based onWolff et al. (2017), though our estimation was limited by the fact that all previous work was in a WM context. One participant did not finish the experiment because they were unwell, and following data inspection, two participants were removed because of poor data quality due to a large number of high impedance channels, and one because of stimulus trigger issues. Thus, EEG-based analyses were conducted based on 29 participants. For behavioural analyses, the first 4 participants were excluded because of missing button press triggers, which, with the further exclusion of the participant who did not complete the experiment, resulted in an analysis of 28 participants (participants with noisy EEG data were included in the behavioural analysis).

Participants were informed about the details of the experiment in advance—including its duration, protocol, and methods—but were left naïve with respect to the purpose and hypotheses associated with the presentation of visual pings. Participants provided their written consent, and after the experiment, they were debriefed and given information about the central manipulation and hypothesis upon request, and they were compensated for their time with £9 per volunteered hour. The study was approved by the ethical committee of the College of Science and Engineering of the University of Glasgow (Application number: 300210113).

### 2.2 Stimulus and apparatus

The presentation of stimuli was controlled using PsychoPy (version 2021.2.3;Peirce et al., 2019) running on Windows 10. Stimuli were presented on a CRT monitor (53.3 cm; 1,024 by 768 pixels) operating at a refresh rate of 60 Hz. Participants were seated in a magnetically shielded room in a chinrest 65 cm from the screen, or at an approximately similar distance from the screen outside the chinrest if they experienced discomfort. Throughout the experiment, a fixation cross (with a visual angle of 0.44°) was presented in the centre of a constantly presented grey background (RGB = 128 128 128; PsychoPy default). All centrally presented stimuli overrode the fixation dot. The visual impulse (i.e., ping) was a single full-contrast bullseye stimulus presented at the centre of the screen for 200 milliseconds (ms; with a diameter of 13° and 0.31° cycles per degree). The ping was generated using MATLAB and edited using GIMP (GNU Image Manipulation Program version 2.10.32).

In the main memory task, participants learned associations between action verbs and images, and were later prompted with the action verb to retrieve the associated image. The action verbs were selected based on usage frequency (largely based onLinde-Domingo et al., 2019) and the image stimulus set was a combination of 192 colour images collated across various royalty free databases, including the Bank of Standardized Stimuli (BOSS;Brodeur et al., 2010), and the SUN database (Xiao et al., 2010). The selected 192 images were constructed to follow a nested category structure of 3 embedded hierarchical levels. At the top level, the set consisted of 96 objects and 96 scenes, which were in turn composed at the middle level of 48 animate and 48 inanimate objects and 48 indoor and 48 outdoor scenes. Moving down to the bottom level, each of the middle level categories branched out into 4 categories (e.g., for animate objects: birds, insects, mammals, and marine animals), each of which contained 12 specific instances (e.g., 12 specific birds). We chose this nested hierarchy of stimulus categories because we did not know a priori what dimension of retrieved memories would be effectively decodable, so we included multiple levels of abstraction and chose one level based on pre-defined criteria (seeSection 2.11). The objects were presented on a white square matching in size to scene images (i.e., the visual degrees of all stimulus categories were 13°). Key presses were registered using a standard QWERTY keyboard.

### 2.3 Procedure

**Fig. 1.**
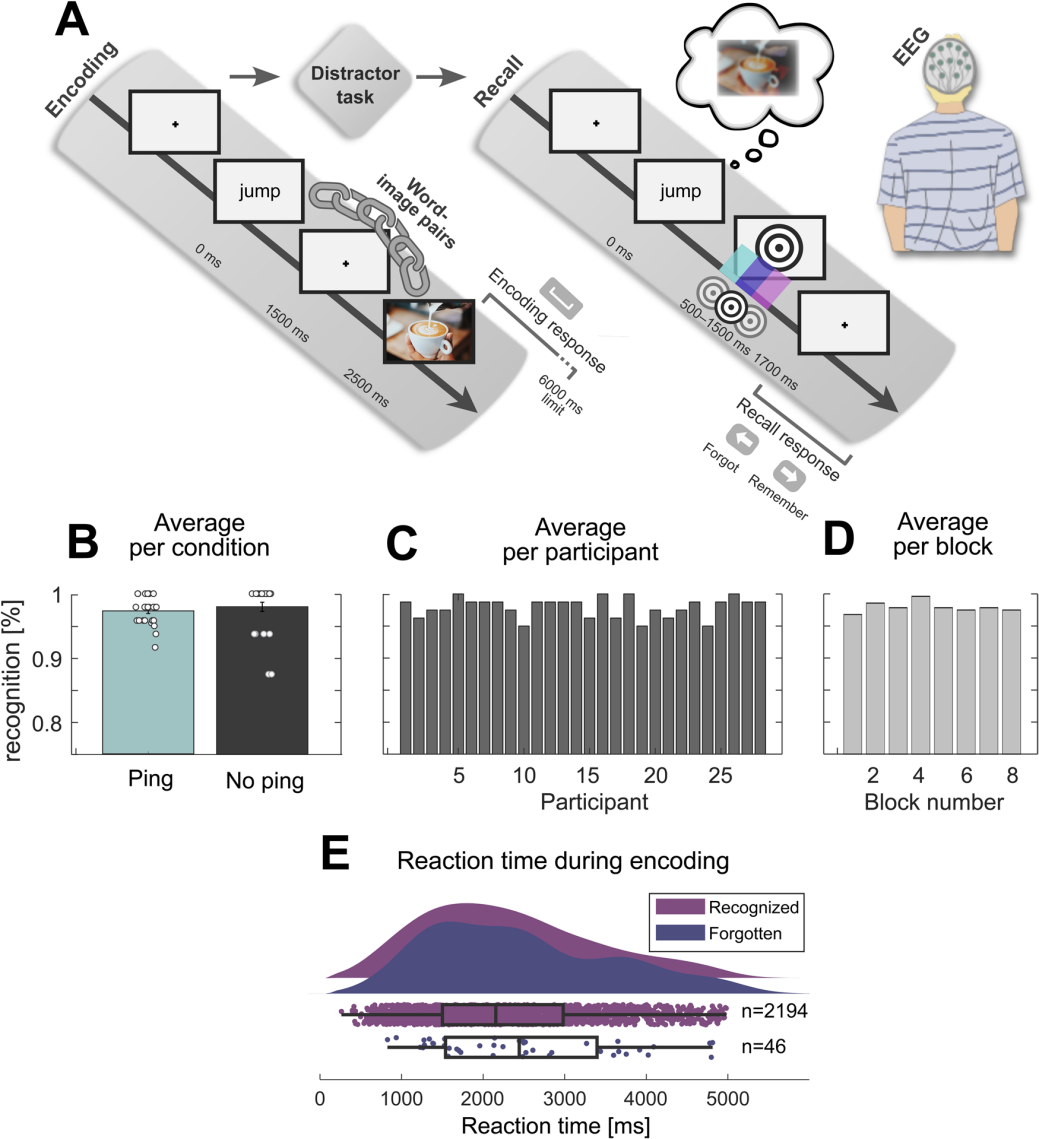
Paradigm and behavioural results. (A) Experimental paradigm. The encoding phase consisted of a word–image pair learning task. This was followed by a distractor task intended to wash out working memory effects. Then, during the critical recall phase, participants were cued with words to retrieve the paired image while visual perturbations (pings) were presented in 75% of trials. In a fourth phase, recognition performance was tested (not displayed). (B) Average performance during the recognition task for trials with and without pings, collapsing across blocks for each participant. Datapoints are individual participants. (C) Average recognition performance per participant (i.e., collapsing blocks). (D) Average recognition performance per block (i.e., collapsing participants). (E) Average reaction time during encoding for subsequently recognized and forgotten trials, collapsing across blocks. Note: in B, C, and D, the y-axis is truncated due to high recognition performance.

The main experiment consisted of eight blocks, each with an encoding, distractor, recall, and recognition phase (Fig. 1A). In total, the main experiment lasted between approximately 45 and 65 minutes depending on the duration of self-paced breaks and electrode impedance maintenance. Before the main experiment, participants were provided with a practice run that covered each phase using example verbs and images that were not used in the main experiment. A standardized set of verbal instructions were provided to guide participants through the practice run. If the participant reported not understanding the task or if they did not give accurate responses, the practice run and instructions were repeated. Then, the main experiment commenced, throughout which EEG was acquired. At the start of each experimental phase, a screen was presented with a reminder of the task instructions and required response keys.

In the encoding phase, participants learned to build a mental association between action verbs and paired images. First, a verb was presented for 1,500 ms (white,*OpenSans*font). Then, after 1,000 ms, the associated image was presented until the spacebar was pressed to indicate the association was encoded (with a 6,000 ms limit). Then, after a 1,000 ms delay, the next verb was presented. During each block’s encoding phase, 10 unique verb–image pairs were learned in one shot. This resulted in 80 encoded pairs across the full experiment, with the images pseudo-randomly selected from the full stimulus set such as to maintain an equal distribution of top-level stimulus categories (40 objects and 40 scenes) and fully random selection over nested middle and bottom levels for each ping and no-ping condition.

The distractor phase that followed was included to flush out WM effects. Here, participants performed an odd–even task lasting 20 seconds. A number between 1 and 99 was presented in the centre of the screen (white,*OpenSans*font), and participants were instructed to press left key for odd numbers, and right key for even numbers. Following a left or right key press, the next number was presented immediately. Participants’ average performance was displayed at the end of the distractor phase, marked as the proportion of correct responses. These data were not further analysed.

Next in each block, the recall phase tested our central manipulation of a ping-based visual perturbation. In this phase, participants recalled the learned verb–image associations of the encoding phase. First, 1 of the 10 encoded verbs was presented for 2,000 ms, serving as the retrieval cue that prompted recall of the associated image. In 75% of trials, a visual impulse was presented in either of three time bins: between 500 and 833.33 ms (“early ping”), 833.34 and 1,116.67 ms (“middle ping”), or 1,116.68 and 1,500 ms (“late ping”) after the onset of the retrieval cue, with a uniform distribution of possible ping times within each bin. This window was chosen on the basis that previous research on cued recall paradigms suggests this is the moment of maximum memory reinstatement (Staresina & Wimber, 2019). In 25% of trials, no visual impulse was presented in order to derive a baseline for statistical hypothesis testing. Participants pressed the left key to indicate that they had forgotten the image associated with the verb cue, or right key to indicate they remembered it. Key presses only resulted in a new trial after 1,700 ms following retrieval cue onset (i.e., 200 ms after the latest possible ping). With presses earlier than that, nothing happened. Participants were given a visual indication that key presses were available via disappearance of the retrieval cue (at its offset; 2,000 ms). During the recall phase, each of the 10 encoded verb–image pairs was tested 4 times, resulting in 40 recall trials per block, and 320 trials in total, comprising 160 objects and 160 scenes. Within participants, each of the four conditions—early, middle, late, and no ping—was configured to present object and scene images equally often (i.e., the top-level stimulus category), with the nested mid- and bottom-level categories randomized. The sequence of presented stimulus level categories, pinging conditions, and verb–image pairs was fully randomized within and across blocks to mitigate order effects. For the within-block randomization, while the 40 recall trials were fully randomized, we ensured the same pair was never recalled twice in direct succession.

Finally, since the cued recall phase only included subjective memory judgements, a recognition phase was included to obtain an objective measure of memory performance for the verb–image pairs. During this 2-alternative forced choice task, 1 of the 10 encoded verbs was presented in the centre of the screen, with 2 images (visual angle of 7.8°) presented underneath, 1 on the left-hand, and 1 on the right-hand side of the screen. Participants chose which of the two images was paired with the central action verb using a left or right key press (with a 5,000 ms time limit). The location of the correctly paired image was randomized between the left and right location. The lure image was always another old image from the immediately preceding encoding phase. Each of the 10 encoded verb–image pairs was tested once in a random sequence. Note that we designed this study to expend most of the available study time on the recall phase to maximize the statistical power of our main analysis, with the recognition phase serving chiefly as a basic check to ensure participants were not skipping through the experiment without memorizing verb–image pairs.

### 2.4 EEG acquisition and pre-processing

The data were recorded using a 64-channel passive EEG BrainVision system (BrainAmp MR; Brain Products) with a sampling rate of 1,000 Hz. For our recording software, we used BrainVision Recorder (Brain Products). The 64 Ag/AgCl electrodes were positioned in accordance with the extended international 10–20 system. Due to a necessary change in the recording system, a different EEG cap type (EasyCap) was used for participants 1 to 14 (subset 1) and 15 to 33 (subset 2). In the first subset, the ground electrode was located on the back of the head, below occipital electrode Oz, and two EOG channels were used to monitor eye movements (placed below and next to the eye; VEOG and HEOG). In the second subset, the ground electrode was on the midline frontal location AFz, and one EOG channel was used to measure eye movements (placed below the eye; VEOG). Furthermore, the cap used in the second subset included channels FT9 and FT10. For event-related potential analyses, we included only electrodes common to both caps to enable a universal visualization of brain activity. Most electrode impedances were kept below 25 kiloΩ, and electrodes with outlier impedances were removed during pre-processing, with their associated data interpolated (see the end of thissection 2.4).

Pre-processing was performed using FieldTrip (Oostenveld et al., 2011) in MATLAB (the MathWorks). First, the continuous EEG data were split up into three datasets: two based on the retrieval phase of the experiment, and one on the encoding phase. For the two retrieval phase datasets, one was created with all trials epoched relative to retrieval cues, and one with trials epoched relative to pings and no-ping (defined by randomly sampling ping times of the pinged trials, yielding so-called pseudo-pings). Put differently, the data were locked once tot=0defined as the retrieval cue, and once tot=0defined as the manipulation of interest or a baseline alternative. For the encoding dataset, which was used for training and testing a decoder on the encoding phase, the event of interest was the image in the learned word–image pair (seeFig. 1A). In each case, the epoched trials were 4 seconds in duration (-1 to 3 seconds relative to the event of interest).

Each dataset was filtered between 0.05 and 80 Hz and downsampled to 250 Hz. Next, bad trials and channels with outlier impedance levels were manually removed via visual inspection. Subsequently, eye movement and muscle artefacts were identified and removed using ICA decomposition, and removed channels were interpolated using spline interpolation (with the FieldTrip function*ft_scalpcurrentdensity*). Finally, the data were re-referenced using a common average and a Laplacian method (current source density), deriving separate data structures for cue-locked, ping-locked retrieval analyses, and encoding analyses.

### 2.5 Behavioural analysis

The experiment was designed to result in high or even ceiling memory performance in order to obtain a maximal number of successfully remembered trials, and to optimally evaluate the central hypothesis of a ping-induced decodability enhancement. We report objective performance for the memory test conducted in the recognition phase, both across pinging conditions (Fig. 1B), participants (Fig. 1C), and across blocks (Fig. 1D). We also report subjective judgements during the recall phase, quantifying how often participants report remembering versus forgetting the word–image pair. Reaction time (RT) during the recall phase is uninformative, because as described in the Procedure section, the response key was locked until 1,700 ms after cue onset, at which point participants likely had already retrieved the associated image (Staresina & Wimber, 2019). Indeed, participants reported actively waiting for response buttons to become available. Thus, we instead analysed RT during the encoding phase as a function of whether the word–image pair was subsequently recognized or not. These RT data were collapsed across participants and blocks (Fig. 1E). For the proceeding analyses, both subsequently recognized and forgotten trials were included.

### 2.6 ERP analysis

For the ERP analyses, only channels common to both electrode cap subsets were used. We applied two types of ERP analyses, one locked to (pseudo-)pings and one to retrieval cues. FieldTrip was used to downsample the data to 250 Hz and a band-pass filter between 0.2 and 40 Hz was used. The data were baseline corrected from -200 ms to 0 ms from events of interest. For ERP traces, we calculated the average activity across posterior channels (C3, C4, P3, P4, O1, O2, Cz, Pz, Oz, CP1, CP2, C1, C2, P1, P2, CP3, CP4, PO3, PO4, PO7, PO8, CPz, POz). For ERP topographies, we used the 61 channels common to both ERP cap types. We statistically evaluated whether pings resulted in higher amplitude ERPs compared with no-ping trials using non-parametric Monte Carlo permutation tests applied to each channel, correcting for multiple comparisons using Bonferroni correction as implemented in FieldTrip, averaging activity from 200 to 400 ms after pseudo-pings (alpha = 0.05; 10^5^randomizations).

### 2.7 Mvpa

We ran three families of MVPA. First, our central analysis comprised an LDA trained and tested on retrieval data (with cross-validation applied, details below). Second, we included a number of analyses that used the encoding period of the experiment, training on encoding and either testing on encoding or retrieval data. Third, we included an LDA trained and tested on retrieval data with spectral power as features.

### 2.8 Main retrieval–retrieval analyses

For the central retrieval–retrieval analysis, all EEG channels available per electrode cap type were used except EOG channels. Depending on the decoded stimulus category level, we trained and tested either a multi-class LDA using FieldTrip (*ft_timelockstatistics*), or a binary-class LDA using the MVPA Light toolbox (Treder, 2020). For our features, we classified the voltage potentials of EEG data re-referenced using a Laplacian transform on the basis that it accentuates local patterns (Kayser & Tenke, 2015). We applied the decoder to both baseline-corrected and non-corrected data to establish the effect of this pre-processing step. Baselines were defined as the 200 ms segment before the orienting event of interest (Wolff et al., 2017), which was the retrieval cue in the cue-locked data, and the (pseudo-)ping in the ping-locked data.

We downsampled the data from 250 Hz to 50 Hz by applying a moving average with a window length of 140 ms, moving in steps of 20 ms. During each step, a Gaussian-weighted mean was applied in which the centre data sample of the window was multiplied by 1, and the tail samples by 0.15 (FWHM = ~81 ms). In a subsequent step, sample by sample, the data were z-scored across channels (i.e., setting every channel to mean = 0 and standard deviation = 1). Classification was then applied to each window, resulting in a decoding time course of 50 samples per second. To evaluate decoder performance, we applied k-fold cross-validation (5 folds, with 25 repetitions). For binary class decoding, we used area under the receiver operating characteristics curve (AUC) as a performance metric because it adjusts for class imbalances (Grootswagers et al., 2017;Xie & Qiu, 2007). For multi-class decoding, where standard AUC is unavailable, we used accuracy and factored in level-specific differences in chance levels. To infer decoding performance values under the null hypothesis, depending on the analysis, we either used no-ping trials or ping trials with shuffled class labels (100 1^st^-level permutations, each with 3 repetitions). These analyses were restricted to the period before button presses were made (i.e., <2,000 ms).

### 2.9 Encoding phase analyses

For the first encoding phase analysis, we trained and tested on encoding data to establish that we can in principle decode stimulus category information from the EEG data. Here, we trained an LDA to derive classifier weights based on the 100 ms to 1,000 ms window of EEG data following each image of the presented word–image pair (henceforth, encoding cue). Then, we tested performance from 500 ms before the encoding cue to 2,000 ms after the encoding cue.

For the second encoding phase analysis, we trained on encoding data and tested on retrieval data, both on ping and no-ping trials, with the data locked to retrieval cues or (pseudo-)pings. We trained an LDA to derive classifier weights based on the 100 ms to 1,000 ms window of data following the encoding cue, which was then used to predict the class labels of retrieval trials during testing. We chose this window because we expected (and also found;Fig. 6) significant category-specific information since participants were processing stimuli present visually on the screen. For both encoding analyses, standard baseline correction was applied as outlined in the main retrieval–retrieval analyses section. Other analysis details were left identical to those of the main analysis.

### 2.10 Spectral features analysis

As will be motivated later on, we ran our central retrieval to retrieval analyses with spectral power as features. Specifically, per electrode, we computed the average power across four frequency bands: theta (3–7 Hz), alpha (8–12 Hz), beta (13–30 Hz), and gamma (35–80 Hz). To do so, the data were Hilbert-transformed (with the FieldTrip function*ft_freqanalysis*). The frequency bands, Hilbert transform, and filter types were adopted from the scripts provided byBarbosa et al. (2021). Other analysis details were left identical to those of the main retrieval–retrieval analysis.

### 2.11 Level selection

We used a multi-class LDA tested and trained on retrieval no-ping trials to determine which retrieved stimulus category (top, middle, or bottom level) is most robustly detectable in the data when our main experimental manipulation was not applied. This level was then locked in for the three families of analyses described above. We selected the level with a high baseline performance to offer a conservative starting point from which we could establish whether pings are a powerful tool to further enhance decodability. However, as we will see in the results, stimulus selection rationales matter minimally because we found no reliable level differences in the no-ping decoder across levels to begin with. For statistics, we performed a Wilcoxon rank sum test comparing the empirical and shuffled decoding performance for each level, in the way described in the next section.

### 2.12 Main analysis

For the statistical analysis of the main hypothesis, we used two-level permutation testing for the ping versus shuffle decodability comparison, and a Wilcoxon ranked sum test for the ping versus no-ping comparison. The former approach, which is based onvan Bree et al. (2022), implemented the following algorithm in pseudo-code—applied window-by-window:

1)  For each 2^nd^-level permutation (10^5^times): Grab one random window-specific decodability value from the 1^st^-level distribution of the 25 permutations of each participant and average the result. This yields 10^5^permuted averages.
2)  Generate one empirical p-value by calculating the percentile of the average empirical decoding value within the distribution of permuted averages.

The latter approach involved taking the Wilcoxon signed-rank test between the distribution of empirical decoder results and 1^st^-level permutation results across participants. We opted for a Wilcoxon test over cluster-based methods because it makes minimal assumptions about the distribution of decoding results (Grootswagers et al., 2017;Wilcoxon, 1945). For both approaches, we adjusted the resulting p-values across windows for their false discovery rate (FDR). Since the p-values are not independent across time, we applied the approach byBenjamini and Yekutieli (2001).

Finally, for ping-locked analyses, we restricted statistical analyses between 0 and 500 ms from ping onset. For analyses locked to retrieval cue, we analysed 500 to 2,000 ms from cue, which is the approximate range where memory reactivation is maximal (Staresina & Wimber, 2019).

### 2.13 Condition-relative decoding peaks

In addition to our main analysis, we carried out a presumably more sensitive analysis to evaluate the possibility of ping-induced decoding enhancements. We reasoned that even if visual pings do not offer an enhancement of LTM decoding performance that is strong enough to emerge in a direct ping-to-no ping or ping-to-shuffle comparison, there could still be a weaker effect that is detectable by factoring in the relative order of decoding peaks across pinging conditions. Specifically, we tested whether trials with an early, middle, and late ping tended to have, respectively, earlier, later, and even later decoding performance peaks. In other words, we tested to what extent decoding peaks captured ping presentation orders (seeLinde-Domingo et al., 2019;Mirjalili et al., 2021for similar peak selection approaches).

First, we took every participant’s stimulus onset asynchrony (SOA)-specific decoding time series—early, middle, and late—and extracted one peak (specified below). Then, we calculated a*peak order distance*(POD) per participant, defined as the absolute serial distance between the order of extracted peaks and true ping presentation order, given by the formula:

$$POD_{non-normalized}=\sum abs\left(peak-true\right).$$

For example, if the decoder peak came first for early ping trials (1−1), third for middle pings trials (3−2), and second for late ping trials (2−3), this would amount to a POD of 2. We divided PODs by the maximum distance (4), normalizing the score between 0 and 1:

$$POD=\frac{\sum abs\left(peak-true\right)}{maximum\;distance}.$$

On this distance metric, lower values indicate a closer correspondence between ping-induced peaks and condition presentation order, which, in turn, confers stronger evidence for ping-based decoding enhancement. For our statistical evaluation, we used a two-level permutation approach (similar tovan Bree et al., 2022). Specifically, we compared the distribution of empirical PODs with PODs calculated across 10^6^second-level permutations, randomly grabbing from the pool of first-level shuffled decoder time courses. The p-values were defined by the resulting percentile of the empirical POD within the distribution of second-level shuffled PODs (one-sided test, empirical < permuted).

For the detection of decoder peaks in this analysis, we detected the maximum peak in the derivative of the cumulative sum of decoding time series. We chose this peak detection method over more standard approaches—such as simply extracting the largest peak from raw decoding series—because independent simulations revealed that this algorithm is most powerful at detecting true POD effects, outperforming a range of competing approaches (Supplementary Materials; Section 2).

## 3 Results

### 3.1 Behavioural results

As expected in light of our experimental design, participants achieved high memory recognition performance, with scores approaching ceiling across behavioural analyses. First, we found no significant difference in memory performance across participants between the ping (M = 0.980, SE = 0.0032) and no-ping condition (M = 0.984, SE = 0.006) during the recognition phase (t(27) = -0.745, p = 0.463;Fig. 1B), suggesting that the decoding analyses that follow are not influenced by absolute inter-condition differences in behaviour. This general near-ceiling performance is also apparent when analysing recognition performance across participants (M = 0.980, SD = 0.015;Fig. 1C) and blocks (M = 0.980, SD = 0.009;Fig. 1D). Furthermore, participants reported a high rate of remembered to forgotten judgements during the recall phase (M = 0.819; SD = 0.022). The average RT during encoding was 2,313 ms for subsequently recognized trials (SD = 1,041 ms; n = 2,194 trials), and 2,472 ms for subsequently forgotten trials (SD = 1,105 ms; n = 46 trials;Fig. 1E).

### 3.2 Event-related potentials

**Fig. 2.**
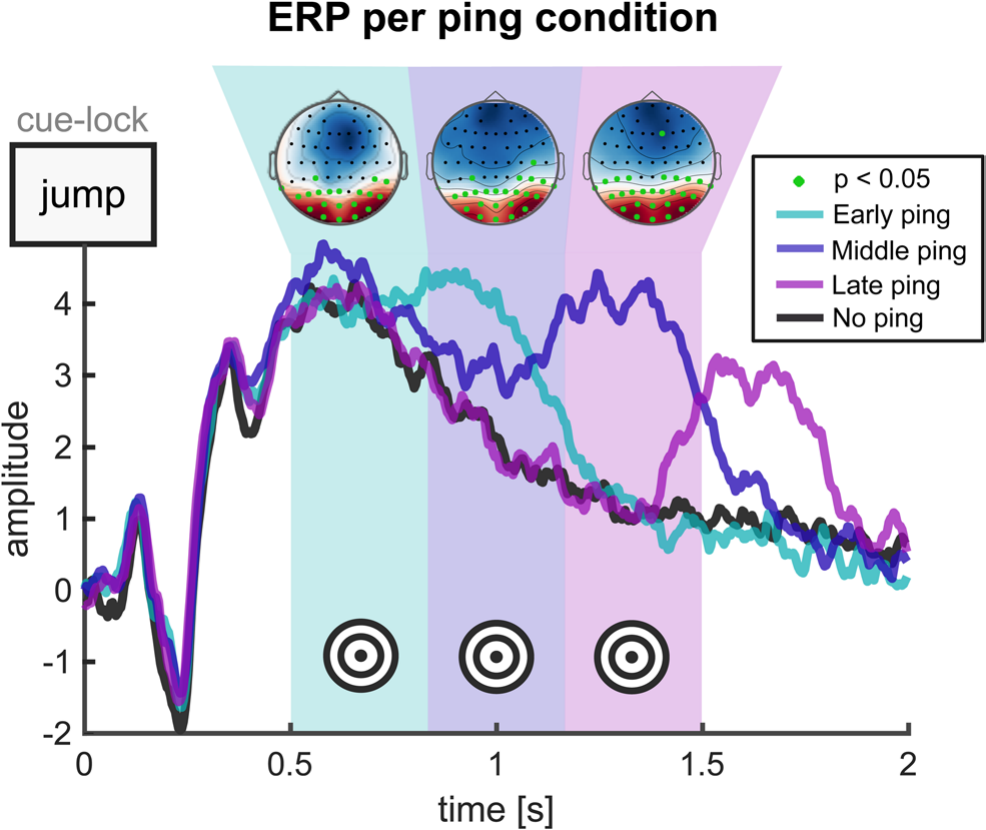
Ping-induced event-related potential. Average evoked response in posterior EEG channels across early (turquoise), middle (blue), late ping (purple), and no ping (black) trials during the recall phase. The inset topographies reveal higher posterior amplitudes following ping trials as contrasted with no-ping trials (Monte Carlo permutation test; Bonferroni-corrected).

We observed a robust evoked EEG response after pings (Fig. 2). Specifically, for each of the three SOA conditions, we observed an extended peak of activity across occipitoparietal channels that followed the distribution of ping times for retrieval cue-locked data, peaking approximately 200 to 300 ms after pings. To further confirm that pings successfully evoked a visual response, we applied a ping-locked analysis across all channels and found significantly higher ERP amplitudes after pinged than no-pinged trials in posterior channels (Fig. 2, insets). Together, the ERP analysis suggests pings yielded a strong time-locked response that could putatively interact with ongoing LTM representations. For cue-locked and ping-locked ERPs for each participant, time-resolved topographical plots, and for p-values of each channel inFigure 2inset topographies, see theSupplementary Materials(Section 1).

### 3.3 Decoding results

#### 3.3.1 Stimulus category selection

**Fig. 3.**
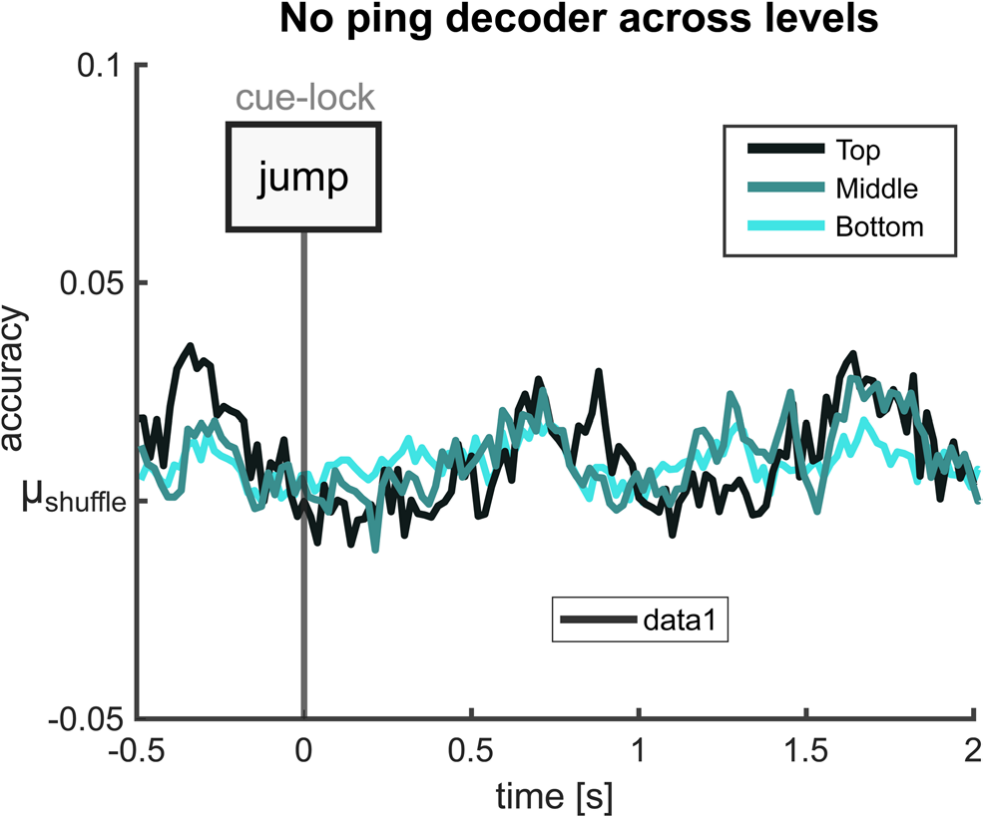
Stimulus category selection. Average decoding accuracy across stimulus category levels (top, middle, bottom). Decoding accuracy was quantified relative to the average performance across shuffled decoding results. No significant differences were observed for any level (Wilcoxon signed rank test, controlled for multiple comparisons using FDR).

We used a multi-class LDA on no-ping trials (25% of the overall recall trials) to determine which retrieved stimulus category (top, middle, or bottom level) is most robustly decodable when our main experimental pinging manipulation was not present (Fig. 3). We found that none of the three levels displayed significant windows of decodability during our retrieval period of interest from 500 to 2,000 ms after cue onset (Wilcoxon signed-rank test; p > 0.11 for top; p > 0.25 for middle; p > 0.07 for bot). We proceeded with the top level, which with its two classes (objects and scenes) afforded simple binary classification with the largest number of trials per class. Next, during our main analysis, we investigated whether pings enhance the decodability of LTM contents.

#### 3.3.2 Main analysis

**Fig. 4.**
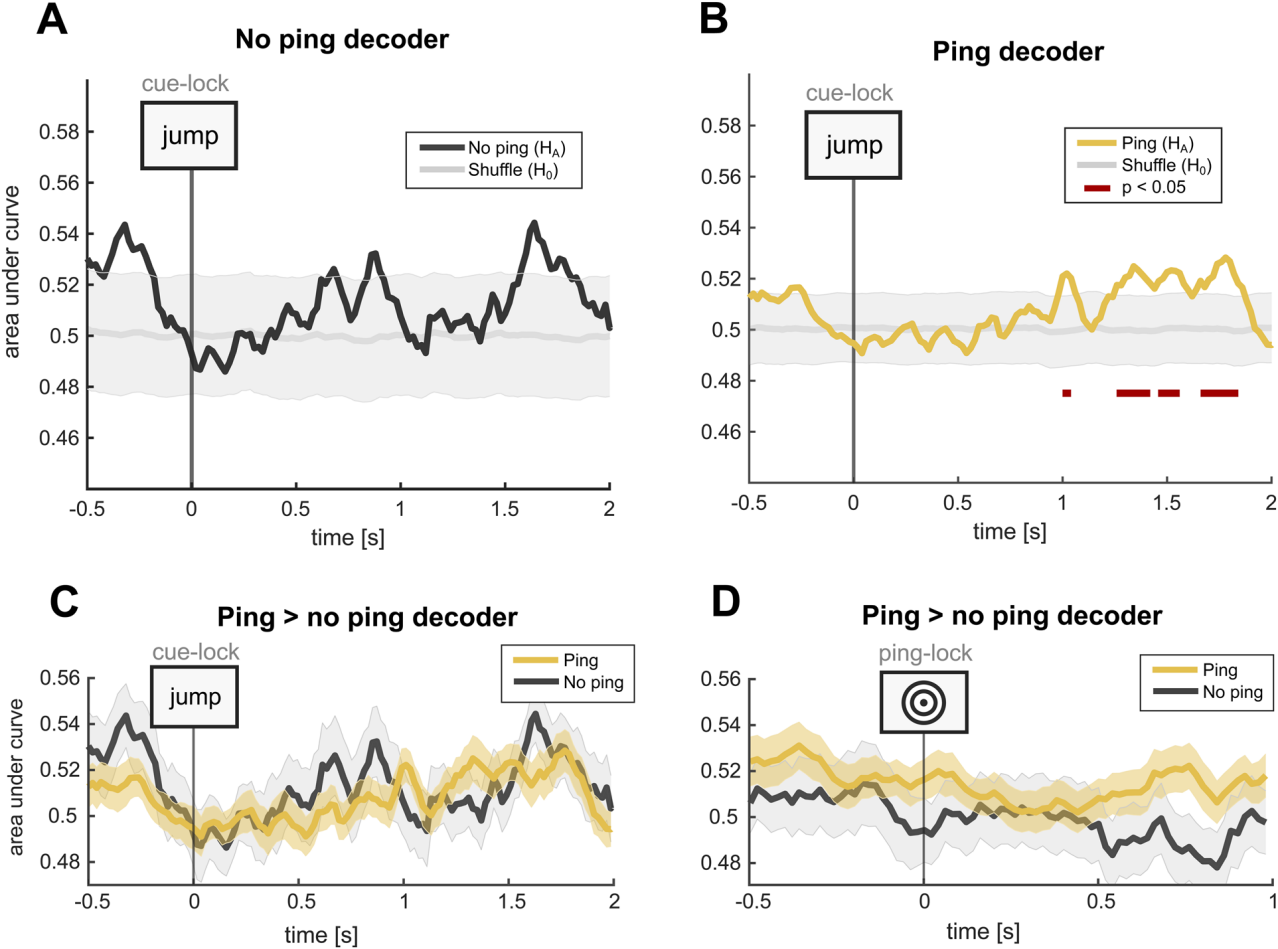
Main decoder analysis. (A) Cue-locked decoding across no-ping trials compared with a shuffled baseline. (B) Cue-locked decoding across ping trials compared with a shuffled baseline. (C) Direct comparison between on ping and no-ping trials. (D) Same as (C), but with the data time-locked to pings and (artificially marked) pseudo-pings. In (A) and (B) the shaded area represents the 5^th^and 95^th^percentile of the distribution of 2^nd^-level permutations of the shuffled decoder, and in (B) and (C) it represents the SEM of the empirical decoder. In (A) and (B), p-values were derived using two-level Monte Carlo permutations, and in (C) and (D) using Wilcoxon signed-rank test (all p-values were corrected using FDR).

For our central analysis, we compared decoder performance between ping and no-ping trials for top-level (objects vs. scenes) classification, both with the data locked to retrieval cues and to pings/pseudo-pings (i.e., artificial markers derived from the pool of ping timings;Fig. 4). For the cue-locked analysis, we found no windows where decoding was above chance for no-ping trials (two-level Monte Carlo permutation; p > 0.49;Fig. 4A), while the ping trials showed several significant windows of content decodability (p < 0.05;Fig. 4B). To validate our analysis, we carried out a direct comparison between the ping and no-ping trial decoder, as opposed to contrasting each condition with a shuffled baseline. In this analysis, we found no evidence for a ping-induced decodability enhancement; neither in the cue-locked (Wilcoxon signed-rank test; p > 0.99;Fig. 4C) nor in the (pseudo-)ping-locked data (p > 0.99;Fig. 4D). Baseline correction does not alter the statistical outcome of these analyses (Supplementary Fig. 5).

In light of an important methodological observation, we place more importance on the latter class of analyses, which directly compares the empirical decoding performance for ping and no-ping conditions without leveraging shuffled results. Specifically, we observed that the standard error of the mean (SEM) of the shuffled distributions varies substantially between ping (μ_SEM_= 0.047) and no-ping (μ_SEM_= 0.028), which we speculated could be explained by trial number differences alone. We inferred that since the ping trial decoder was trained and tested on three times more trials than the no-ping trial decoder, this might naturally shrink SEM values of the shuffled distribution and thereby modulate test statistics. In support of this interpretation, we built a simulation which confirms that an increase in the number of trials (and the number of decoding classes) reduces p-values, but only if there is an effect in the data (Supplementary Materials; Section 3). Therefore, instead of relying on ping-to-shuffle and no-ping-to-shuffle comparisons where power differences might misleadingly lead us to infer a ping-related enhancement, we placed most credence in the direct comparison between ping and no-ping trials in which shuffled results are sidestepped (Fig. 4C, D; see theSupplementary Materialsfor an extended discussion; Section 3.3).

#### 3.3.3 Condition-relative decoding peaks

**Fig. 5.**
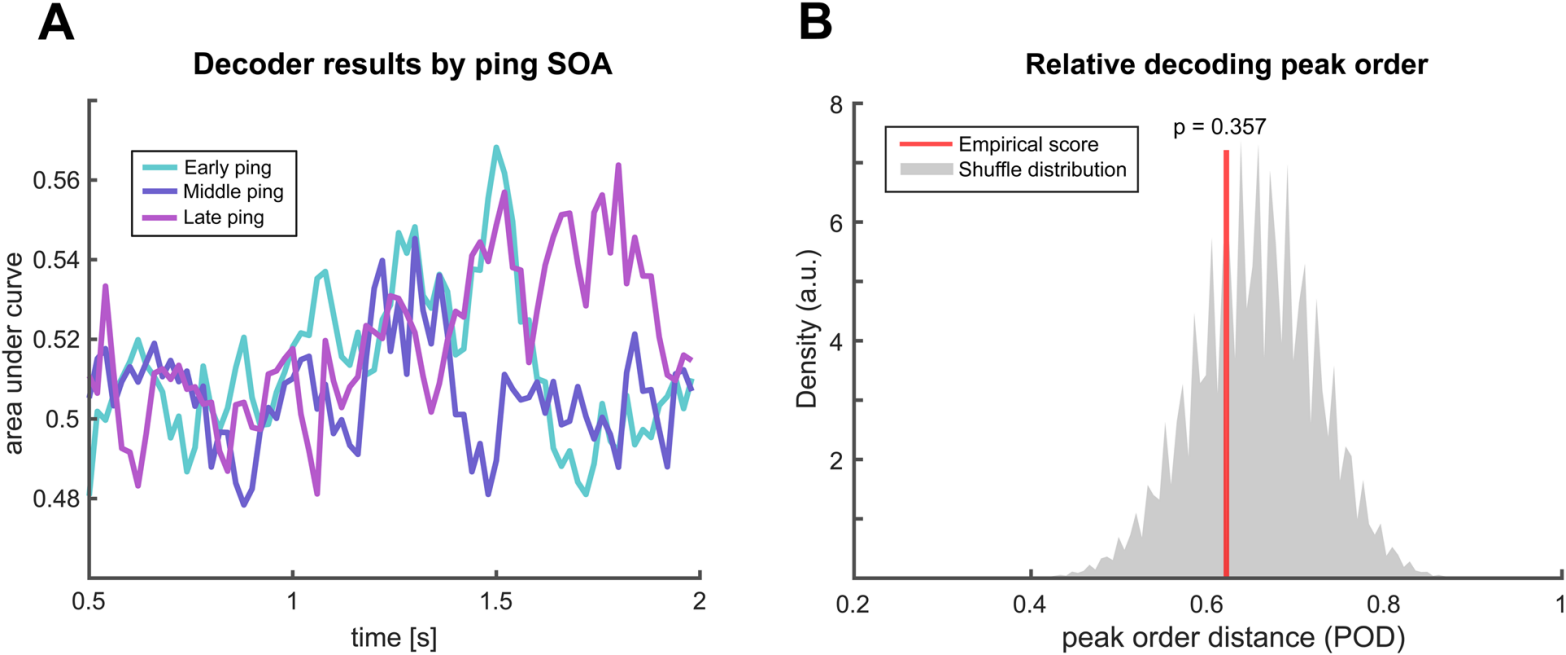
Condition-relative peak analysis. (A) Decoding results specific to for early (cyan), middle (blue), and late (purple) ping conditions, averaged across participants. (B) Peak order distance scores for the empirical decoder (red line) among a pool of 2^nd^-level permutations derived from the shuffled decoder (grey distribution).

Next, we turn to the presumably more sensitive peak-order analyses. Qualitatively, we observe no ordered structure in decoder peaks when averaging across participants for each SOA pinging condition (Fig. 5A). For a quantitative analysis, we formally compared peak order structure by comparing POD scores for the empirical and shuffled decoder using two-level permutation tests. This analysis confirmed the previous result by revealing no significant evidence for the hypothesis that pings induce systematic differences in the order of decoding peaks (p = 0.357;Fig. 5B).

#### 3.3.4 Training on encoding

**Fig. 6.**
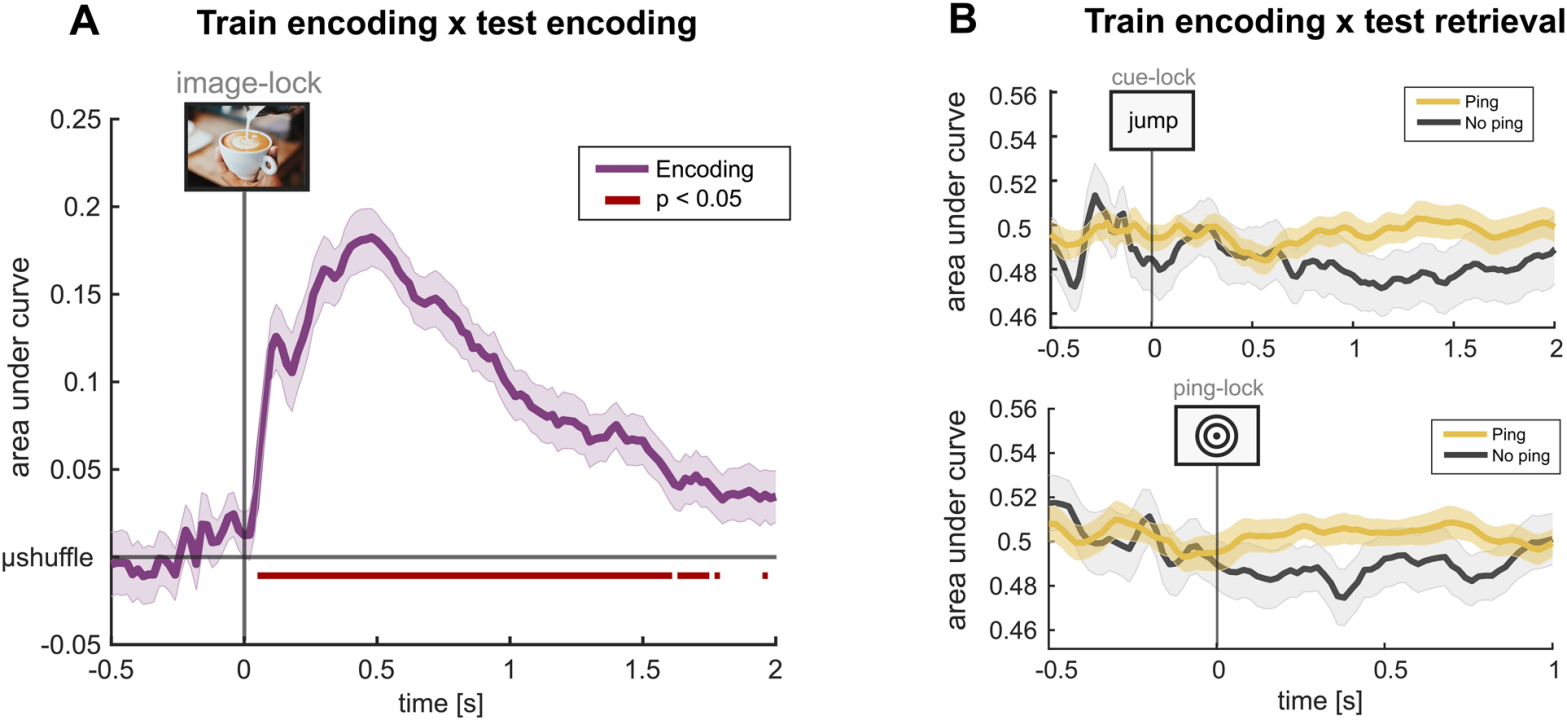
Encoding analyses. (A) Decoding results when training and testing a classifier on the encoding phase of the experiment for the top-level stimulus category. (B) Decoding results for the top level when training on encoding data and testing on retrieval data, either locked to cue (top row) or pings (bottom row). In (A), binary decoding accuracy was quantified relative to the average performance across shuffled decoding results, and p-values were obtained as described for the stimulus category selection analysis. In (B), p-values were derived as described for the analyses associated withFigure 4C and D.

One possibility is that using the encoding phase of the experiment to train a classifier optimizes the detection of stable neural signatures of object category given that stimuli are visually presented. In general support of this, decoding object category during visual perception achieves reliable and high classification performance (Cichy & Pantazis, 2017). Indeed, we found a strong decoding peak when training and testing in the encoding phase of the experiment (Fig. 6A; Wilcoxon signed-rank test; p < 0.05 for timepoints marked inFig. 6A). This confirms that the decoding method and acquired EEG data are adequate and offers an alternative avenue for the training of classifier weights. Does decoder training on the encoding segment and testing during the retrieval phase show evidence of a ping-induced classification performance enhancement? Bolstering the interpretation that pings do not reliably improve classification, we observe no significant increase in performance following pings compared with no pings; neither in cue-locked (Fig. 6B; top row) nor in ping-locked analyses (Fig. 6B; bottom row; Wilcoxon signed-rank test; p > 0.99).

#### 3.3.5 Training and testing on spectral power

**Fig. 7.**
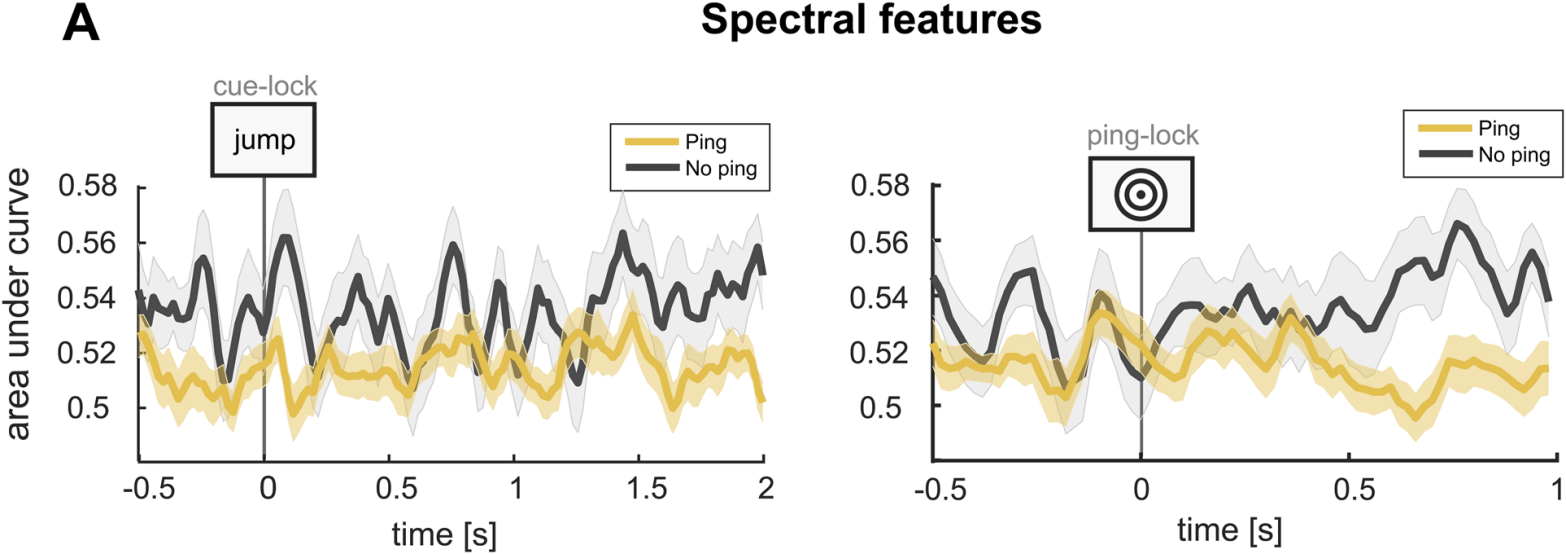
Decoding analyses spectral features. (A) Decoding results with spectral power in theta, alpha, beta, and gamma bands as features. The decoder was applied to the retrieval phase of the experiment for the top-level stimulus category. The p-values were derived as described for the analyses associated withFigure 4C and D.

Another possibility is that alternative feature extraction methods yield ping-induced classification enhancements. In support of this,Barbosa et al. (2021)re-analysed a working memory dataset byWolff et al. (2017)and observed that using power values can reveal significant decoding during segments where voltage potentials did not. Second,Mirjalili et al. (2022)systematically evaluate the effects of various classification parameters and show that power values can produce robust decoding performance in an episodic memory task. Thus, we reran our central analyses with spectral power in theta, alpha, beta, and gamma frequencies per electrode as the classifier features rather than voltage potential per electrode. In contrast toBarbosa et al. (2021), we found that in this long-term memory study, the use of spectral power as features does not uncover evidence for a ping-induced enhancement of decoding performance (Fig. 7A; Wilcoxon signed-rank test; p > 0.99), underscoring the running null results interpretation.

## 4 Discussion

In this study, we set out to systematically evaluate visual perturbation, or ping-based stimulation, as a method to dynamically enhance the decodability of reactivated neural representations during memory recall. Such an approach could supplement offline analytical approaches by adding further read-out enhancements online at the experiment side. Despite promising results in the WM literature, in this LTM context we found no evidence for a ping-based enhancement across several time-resolved decoding analyses. While pings evoked a strong brain response, they did not detectably boost neural signatures of memory representations in EEG data. We draw this conclusion based on two key results. First, in the main comparison between pinged trials and non-pinged trials, we found no significant decoding difference regardless of whether the data were locked to (pseudo-)pings or retrieval cues. Furthermore, across additional analyses which (i) leverage the constraining information of ping presentation timings, (ii) train on the encoding segment of the task, or (iii) use spectral power as features, we also found no evidence for ping-related decoding boosts.

There are three overarching explanations for these null results. First, there could be an effect in the data that was left undetected analytically or statistically. Second, there could be an effect that manifests across other experimental contexts, but not with this study’s parameters. Third, there could be no effect in principle, with LTM-based retrieval eluding the enhancement of representational readouts using pings. We consider each option in turn.

First, the signal analysis parameter space is high, with variability in parameters across pre-processing and statistical analysis steps potentially altering the results. One important source of variability concerns the implementation of decoding techniques. Namely, we do not rule out that untested decoding methods such as linear approaches beyond LDA or non-linear classifiers would have resulted in performance enhancements induced by pings. More trivially, our analyses could have been optimal, with our key statistical results containing a type-II statistical error.

Second, the parameter space on the experimental side is also high. Here, we opted for a word–image association task, which has previously been shown to afford classification-based inferences about memory processing in the brain (Kerrén et al., 2022;Linde-Domingo et al., 2019;Martín-Buro et al., 2020;Mirjalili et al., 2021). However, other LTM tasks might be better suited to reveal ping-based enhancements. Besides the memory task itself, a key set of parameters concerns the presentation of pings. In this study, we chose a high-intensity, short-lasting ping presented with a uniform distribution between 500 and 1,500 ms after retrieval cues. This time window was selected based on a review of the timeline of memory reactivation during cued recall, which suggested a maximal content reinstatement within this period (Staresina & Wimber, 2019). However, we observed that decoding was highest late within and even after this range, at approximately 1,200–2,000 ms after cue (seeFig. 4D). Decoding plateaus that exceed 1,500 ms have also been observed in recent work that employed a similar task and analysis pipeline (Kerrén et al., 2022). This raises the possibility that the aforementioned 500 to 1,500 ms window is biased to be too early—perhaps because it was estimated based on intracranial EEG research where recordings tend to focus on the hippocampus and other regions that activate early during retrieval (Merkow et al., 2015;Mormann et al., 2005;Staresina et al., 2019). Put differently, it is possible that we did not find significant effects because the signatures of retrieved contents tended to arise robustly only after our ping presentation times. We recommend that future work considers later ping times, potentially informed by maximum decodability periods found in this and other work, or ideally in newly acquired pilot data. Finally, one possible experiment-related explanation for these null results is that the LTM trace for word–image pairs was drowned out quickly by other neural processes, such as the initiation of memory codes associated with behavioural responses and metacognitive evaluations.

Moreover, additional research could explore parameters such as ping duration, intensity, and strength. In this study, we used a single high-contrast bull’s eye stimulus presented for 200 ms, which has resulted in ping-based WM decoding enhancements (Ten Oever et al., 2020). Still, other parameters might work better—for example, the study byWolff et al. (2017)presented three adjacent bull’s eyes for 100 ms. To offer some support for the suboptimal nature of our pinging parameters, a supplementary analysis suggests that pings failed to reduce the overall variance of brain activity (Supplementary Materials; Section 4;Supplementary Fig. 8). This raises the possibility that our manipulation failed to target our proposed mechanisms for signal-to-noise ratio enhancement, which is the reduction of intertrial variability of neural activity underpinning long-term memory. However, this analysis constitutes a weak evaluation of this possibility because it evaluates all neural dynamics, not the finer-grained patterns associated with LTM specifically.

A final point on experimental parameter space is that besides visual pings, a plethora of other perturbational approaches are on stock that could realize the ping’s proposed effects. Also inspired by WM research, stimulation using auditory impulses might offer a multimodal route to improving the readout of LTM contents (Kandemir & Akyürek, 2023). Furthermore, brain stimulation methods such as transcranial magnetic and ultrasound stimulation have the potential to regularize brain activity through the induction of a dynamics-altering magnetic or ultrasound pulse (Moliadze et al., 2003;Mueller et al., 2014).

A third possibility is that none of these factors explain our null results, with ping-based approaches restricting their utility to WM tasks. One specific possibility could be that WM and LTM differ in their mechanisms of action, with separate kinds of neural processes underpinning them. Indeed, classically WM is believed to involve the active maintenance of stimulus-induced information (Fuster & Alexander, 1971;Goldman-Rakic, 1995), whereas LTM is assumed to be based on a generative reconstruction of past experience based on the activation of silent information-storing engrams (Josselyn & Tonegawa, 2020). Perhaps the sweep of activity associated with the ping interacts more effectively with functional brain activity maintained continuously from stimulus onset, thus explaining WM-to-LTM differences. Speaking against this interpretation is work that suggests WM representations are encoded in activity-silent networks through short-lasting synaptic changes (Kamiński & Rutishauser, 2020;Masse et al., 2020;Stokes, 2015), which would not be fundamentally different from LTM works, at least at shorter timescales as used in the present study. Contradicting this, in turn, is a critique which argues that evidence for activity-silent networks in WM tasks could alternatively be explained by LTM processes kicking inBeukers et al. (2021). Furthermore, at the intersection of the first and third possibility, WM studies in which ping-based enhancements have been shown usually involve the classification of simple stimuli such as gratings, which may be maintained in sensory regions where pings are also processed. In this study, participants retrieved complex images with a semantic component, which could move the relevant representations to higher-order areas insufficiently influenced by ping-based activity. Overall, since it is unclear to what extent the mechanisms of WM and LTM differ, to what extent WM and LTM intertwine in studies where ping-based effects have been demonstrated, and even to what extent the current paradigm activates WM versus LTM processes, we avoid firm interpretations in this part of the possibility space. In summary, although pings unambiguously elicited expected patterns of visual activity (Fig. 2), we failed to find effects on memory decoding, either because they were left undetected in our analysis, because they do not show up in our experimental protocol, or because they do not exist.

This study builds on decoding research that investigates the physical basis of memory, leveraging its findings for a strictly instrumental purpose: the systematic enhancement of LTM readouts. This undertaking is key because the field presently lacks temporally sensitive neuroimaging methods that enable the consistent and clear readout of memory representations, which is needed to explain how the brain implements memory processes. Furthermore, the analytical challenges, null results, and possible solutions considered in this work could inform practice in fields closely aligned with memory, such as the neuroscience of mental imagery (Dijkstra et al., 2018).

To conclude, most efforts to improve memory readouts from electrophysiology data have been restricted to the signal analysis end. Here, we advocate for research that explores online manipulations as memory tasks are unfolding, which has previously shown to complement or synergize with decoding techniques. For long-term memory decoding in particular, however, such interventions are scarce, which limits research because memory involves low decodability to begin with. Thus, even if a further carving out of the parameter space does not demonstrate a notable benefit of visual perturbations, future research should creatively explore alternative online methods such as multimodal stimulation and non-invasive brain stimulation.

## Supplementary Material

Supplementary Material

## Data Availability

Pre-processed EEG data and behavioural data are available athttps://doi.org/10.5281/zenodo.13909754, and the code to run and analyse the experiment is available athttps://github.com/sandervanbree/MemPing/.
