# Supplementary material for: No evidence that visual impulses enhance the readout of retrieved long-term memory contents from EEG activity
Source: Imaging Neurosci (Camb). 2024 Oct 24;2:imag-2-00330. doi: 10.1162/imag_a_00330 (PMC12290828; doi:10.1162/imag_a_00330)
Supplement: Supplementary Material [file imag_a_00330-supp.pdf]

Supplementary Materials

1. Event-related potential

Retrieval cue-locked ERP across all trials (posterior channels)

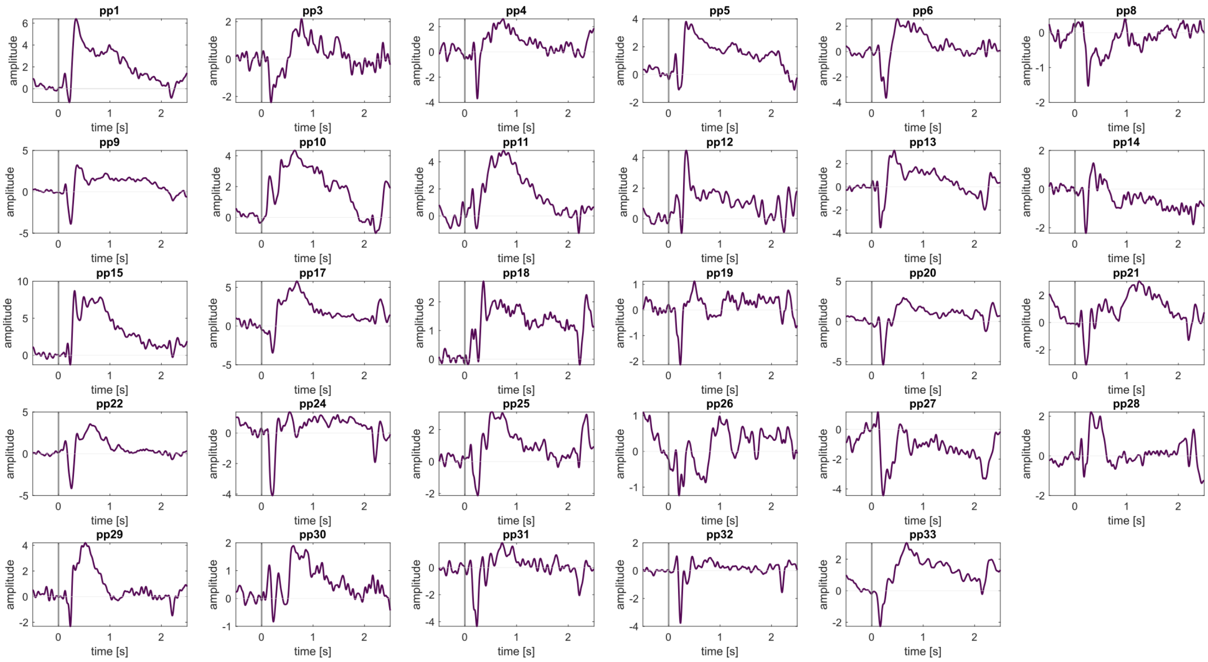

Supplementary Figure 1. Retrieval cue-locked ERP. The purple trace reflects the average cue-locked response for each participant across posterior EEG channels. The grey horizontal line represents cue onset. For more details, see the Methods section in the main text. The amplitude on the y-axis is in arbitrary units.

Retrieval ping-locked ERP across all trials (posterior channels)

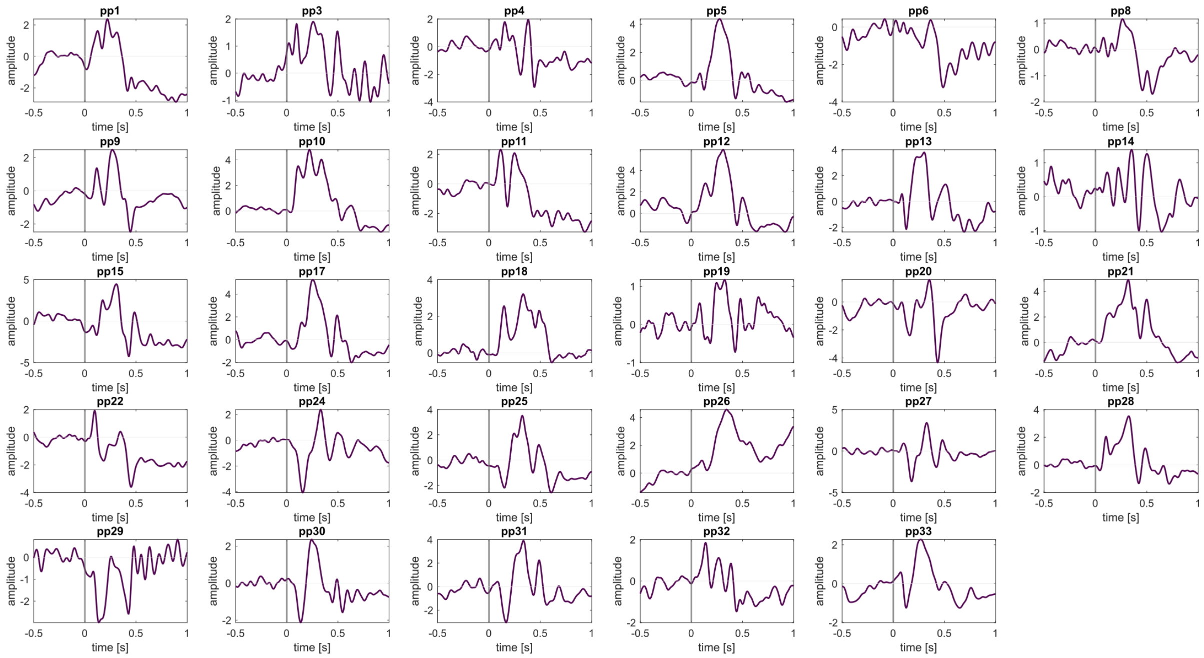

Supplementary Figure 2. Retrieval ping-locked ERP. The purple trace reflects the average ping-locked response for each participant across posterior EEG channels. The grey horizontal line represents ping onset. For more details, see the Methods section in the main text. The amplitude on the y-axis is in arbitrary units.

Retrieval cue-locked topographies across all trials and participants

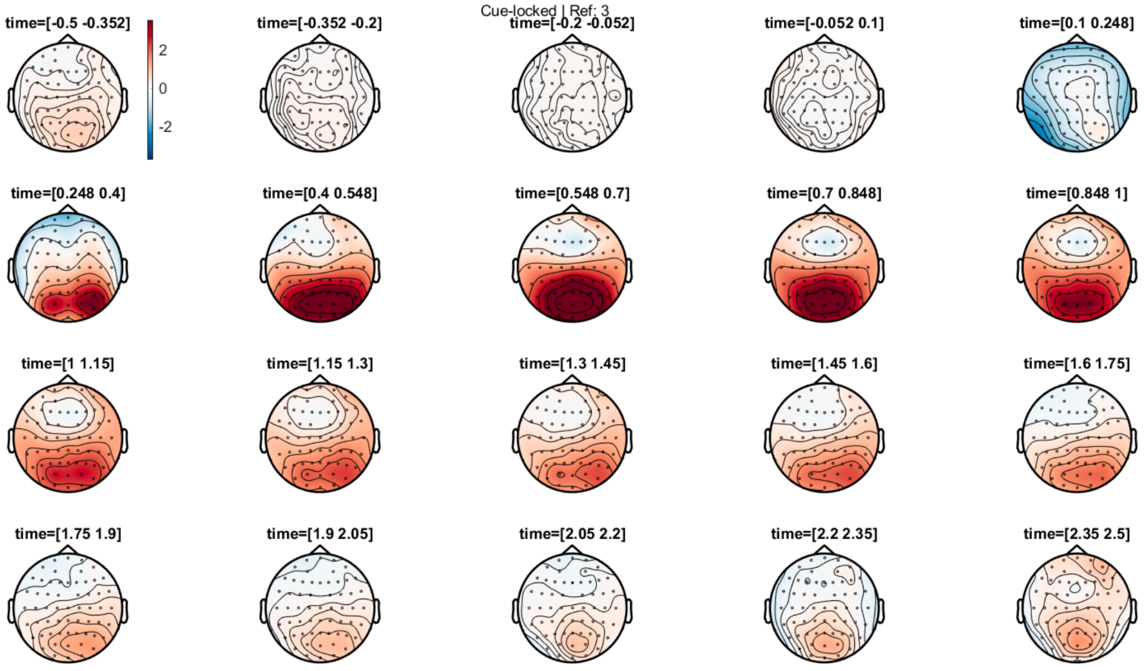

Supplementary Figure 3. Retrieval cue-locked topographies. These topographical plots represent the average cue-locked activity across participants. The colours represent the difference in EEG activity before and after cue onset in arbitrary units (red colours represent $activity_{post}$ > $activity_{pre}$ and vice versa for blue colours). No statistical analysis was carried out for these topographical contrasts. For more details, see the Methods section in the main text.

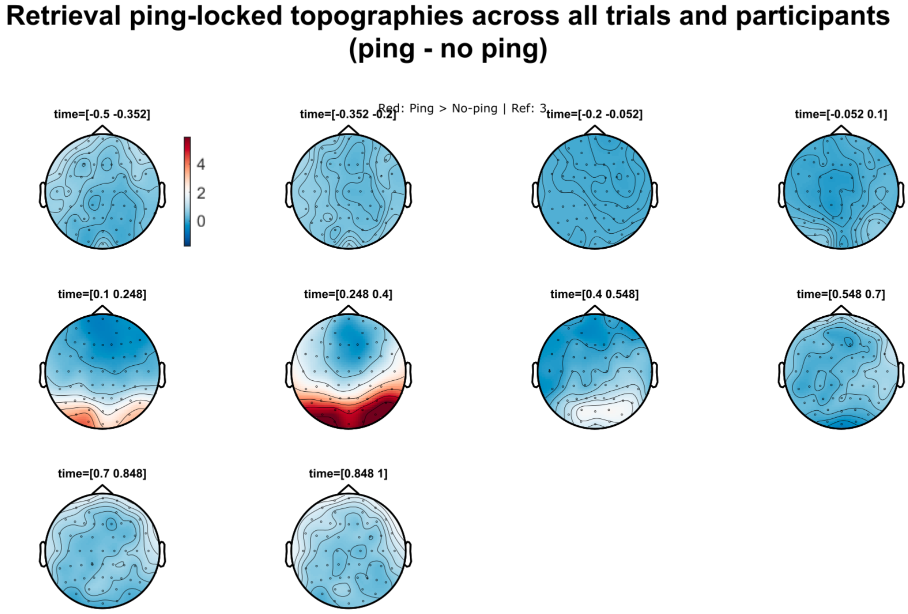

Supplementary Figure 4. Retrieval ping-locked topographies (ping vs. no ping trials). These topographical plots represent the average cue-locked activity across participants. The colours represent the difference in EEG activity between ping and no-ping (red colours represent $activity_{ping}$ > $activity_{no\ ping}$ and vice versa for blue colours). No statistical analysis was carried out for these topographical contrasts. For more details, see the Methods section in the main text.

Baseline correction

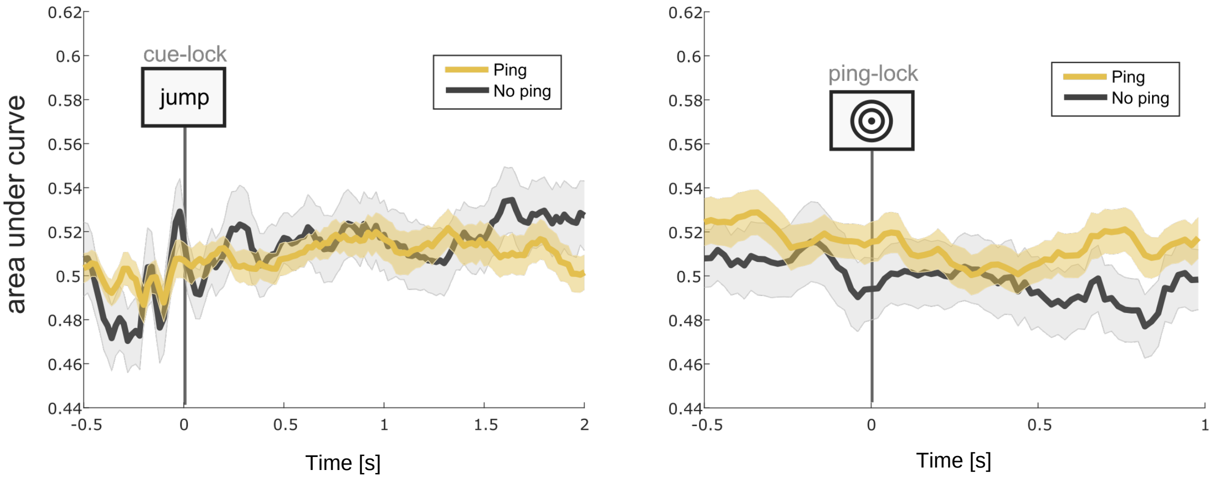

Supplementary Figure 5. Decoding results with baseline correction. We applied baseline correction from -200 ms to 0 ms from the event of interest; i.e., retrieval cues in the cue-locked data and pings or pseudo-pings in the ping-locked data. All other analysis parameters were identical to the analyses presented in main Figure 4. Since the application of baseline correction does not produce significant decoding (Wilcoxon signed-rank test; $p > 0.99$), this suggests that this preprocessing step does not explain our null results.

Channel	Early ping (p-val)	Middle ping (p-val)	Late ping (p-val)
Fp1	0.032	0.616	0.246
Fpz	0.089	0.079	0.011
Fp2	0.042	0.537	0.115
AF8	0.119	0.422	0.318
AF7	0.014	0.272	0.954
AF3	0.439	0.23	0.123
AF4	0.712	0.541	0.014
F7	0.002	0.346	0.358
F5	0.068	0.439	0.33
F3	0.119	0.477	0.693

F1	0.597	0.662	0.119
Fz	0.053	0.551	0.003
F2	0.013	0.473	0
F4	0.341	0.939	0.049
F6	0.427	0.559	0.707
F8	0.131	0.826	0.825
FT8	0.001	0.097	0.049
FC6	0.177	0.142	0.78
FC4	0.962	0.176	0.881
FC2	0.245	0.276	0.09
FC1	0.969	0.503	0.881
FC3	0.176	0.279	0.28
FC5	0.011	0.083	0.112
FT7	0.002	0.298	0.127
T7	0.004	0.047	0.043
C5	0.013	0.027	0.051
C3	0.002	0.022	0.01
C1	0.148	0.049	0.04
Cz	0.144	0.155	0.114
C2	0.305	0.182	0.104
C4	0.02	0.004	0.014
C6	0.003	0	0.003
T8	0.002	0.002	0.003
TP10	0.002	0	0
TP8	0	0	0
CP6	0	0	0
CP4	0.001	0	0.001
CP2	0.006	0.001	0.001
CPz	0.019	0.006	0.01
CP1	0.272	0.002	0.003
CP3	0	0	0.001
CP5	0.002	0.001	0.001

TP7	0.002	0.008	0.001
TP9	0	0	0
P7	0	0	0
P5	0	0	0
P3	0	0	0
P1	0	0	0
Pz	0.001	0	0
P2	0.001	0	0
P4	0	0	0
P6	0	0	0
P8	0	0	0
PO8	0	0	0
PO4	0	0	0
POz	0	0	0
PO3	0	0	0
PO7	0	0	0
O1	0	0	0
Oz	0.001	0	0
O2	0	0	0

Supplementary Table 1. P-values associated with inset topographies in main text Fig. 2; rounded to three decimal points.

2. Peak order analysis simulation

2.1 Time series simulation

We used MATLAB (the MathWorks) to generate time series with two components: (1) a peak at a fixed time point (1000 ms), and (2) autocorrelated noise generated using a random walk procedure. We matched several characteristics of the simulated time series to our empirical decoding data, including the analysis period (500 to 2000 ms), sampling rate (50 Hz), and the number of (virtual) participants (N = 29). The signal-to-noise (SNR) ratio of the simulation was set to 1.15, qualitatively matching peaks observed in the empirical data. We found that varying the SNR does not significantly

alter the results. We generated 1000 trials per participant, resulting in 29000 trials in total.

2.2 Analysis

We included a smoothing parameter that implemented one of four smoothing methods: no filter, a Gaussian filter, a Savitzky-Golay filter, and a median filter. We also included a window size for smoothing, set to 10 samples for our main analysis. We compared the performance of eight peak detection methods, evaluating each of them based on the absolute distance between estimated peaks and true peaks—amounting to a simplified version of the *peak order distance* score described under *condition-relative decoding peaks* in the main text. The winning method was locked in for our empirical analysis. We tested eight peak detection methods:

(1) Low-pass approach, where the maximum peak was computed after a low-pass filter was applied to the time series.
(2) Maximum value approach, which simply computed the maximum value per time series regardless of whether the surrounding data was peak-like.
(3) Cumulative sum approach, which computed the maximum peak in the derivative of the cumulative sum of the data.
(4) Cumulative integral approach, which computed the maximum peak in the cumulative integral of the data via the trapezoidal method.
(5) Integral cumulative sum approach, which worked as the previous method but which operates over the cumulative sum rather than raw time series.
(6) Wavelet transform-based method, which finds the maximum peak in a wavelet decomposed version of the data.
(7) Hilbert transform-based method, which find the maximum peak in the amplitude fluctuations in the envelope of the time series.
(8) Cross-correlation method, which finds the time lag with a maximal correlation between the signal and iteratively shifted versions of itself.

2.3 Results

We found that approach 5—the integral cumulative sum approach—reliably achieves low absolute distance errors across parameters (Supplementary Figure 6). These results were generally unchanged across adjustments of the parameters (to evaluate

this, we refer to the code published with this manuscript). Thus, we used approach 5 in our main peak order detection analysis.

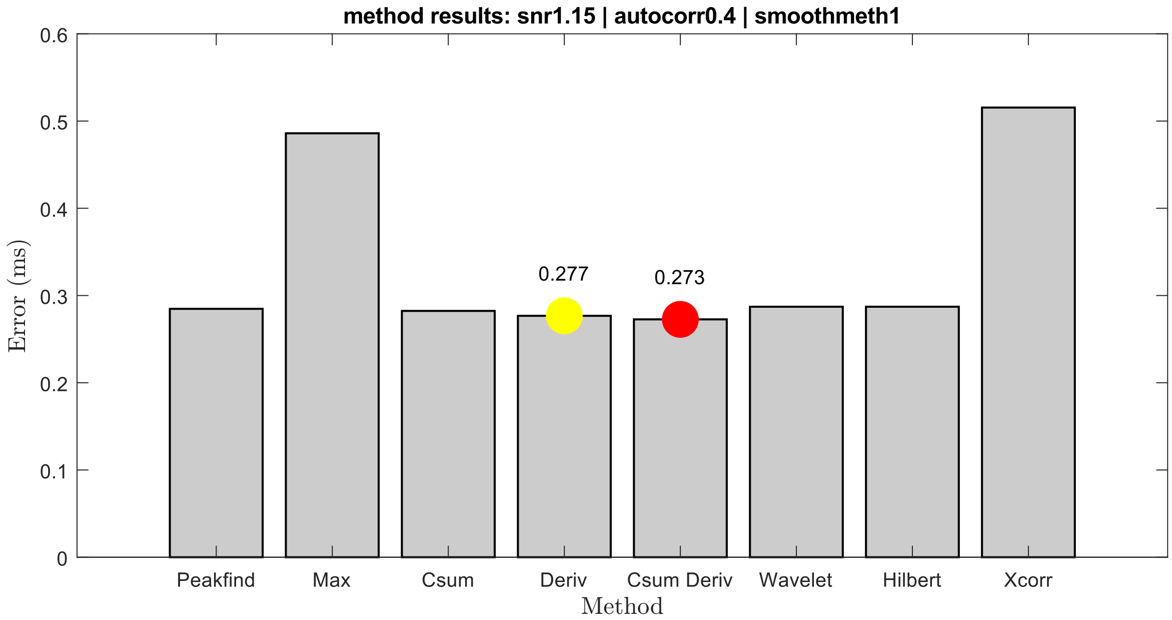

Supplementary Figure 6. In simulated time series, the integral cumulative sum approach works best for detecting a peak in noisy time series. The red circle indicates the best-performing method, and yellow the second best-performing method. Errors were computed based on the absolute distance in milliseconds (ms) between estimated and true peak location.

3. Class and trial number decoding simulation

We speculated based on a qualitative inspection of the empirical decoding results that the number of trials (N_{trials}) and classes ($N_{classes}$) reduces the statistical significance of decoding results. We evaluated this intuition by demonstrating using simulations that these two parameters do indeed influence the variance of shuffled and empirical results, which in turn affects p-values but only if there is a true effect in the data.

3.1 Time series simulation

Using MATLAB, we generated one ground truth vector of class labels which represented the true class structure in the simulated data. This vector contained a random sequence of integers randomly grabbed between the interval 1 and $N_{classes}$. For example, with 16 classes, the ground truth pattern might have contained a sequence of [2,7,15,4,13,17] and with 2 classes a sequence of [2,2,1,2,1,2].

Then, to simulate shuffled decoding results, we generated a distribution of random sequences of integers identical to the ground truth procedure, but with newly generated random integers. These random sequences represented shuffled decoding results and were scored based on their average element-wise correspondence to the ground truth pattern—which is how decoding accuracy is normally computed. For example, if the permuted vector is [2,1,2,2,1,1] and the true sequence is [2,2,1,2,1,2], the accuracy would be 50% because half of the class labels correspond to the true structure. Trivially, with increasing repetitions the shuffled distribution will approach chance level predictions of the ground truth pattern (i.e., the expected value is exactly at $1/N_{classes}$).

Finally, to simulate empirical decoding results, we again generated a distribution of random integers identical to the procedure for shuffled and ground truth decoding results. However, for these data we manually injected between 0% and 60% of the ground truth pattern into the otherwise random vector, effectively modulating decoding accuracy. With 0% of the ground truth injected, there is no statistically detectable difference in accuracy between empirical and shuffled decoding results, because the vectors are equally random. With 60%, the encoding results are substantially more accurate than shuffled results, yielding above chance decoding accuracy.

We simplified our simulation by operationalizing the variable N_{trials} as the number of elements in the vector, allowing us to efficiently investigate how the number of observations influences statistical tests. We also compared $N_{classes} = 2$ and $N_{classes} = 16$, which respectively match the number of classes for top- and bottom-level category decoding in our main experiment. Both N_{trials} and $N_{classes}$ were independently manipulated in a $2 * 2$ factorial design, allowing us to evaluate the contribution of each variable toward statistical outcomes (as a function of effect size).

3.2 Results

First, with respect to $N_{classes}$, we found that increasing the number of classes reduces the spread of both shuffled and empirical decoding results (Supplementary Figure 7; columns). This happens both if there is no true effect in the empirical data, and when a significant proportion of the ground truth is inserted into the empirical data. Second, we found that N_{trials} similarly reduces the variance of both shuffled and decoding results, both across low and high $N_{classes}$ (Supplementary Figure 7; top and bottom

half). Thus, we conclude that both factors modulate the likelihood of finding a significant difference between empirical and shuffled results, but only if there is a true effect in the data. Indeed, as we can glean from the results based on non-existent effects, the distributions of empirical and shuffled will overlap regardless of N_{trials} or $N_{classes}$ (Supplementary Figure 7; left half). In contrast, if there is an effect (60% injected ground truth), both N_{trials} and $N_{classes}$ independently increase the distributional distance between empirical and shuffled accuracy values.

3.3 Discussion

We found that N_{trials} and $N_{classes}$ independently reduce the variance of accuracy results, which will affect statistical tests between empirical and shuffled distributions but only if there is an effect in the data. As suggested in the main text, these findings suggest that statistical analyses that depend on variance comparisons between empirical and shuffled distributions should be interpreted with care if it is done across conditions with varying N_{trials} and $N_{classes}$. With regard to our main analysis for example, the fact that the decoder based on pinged trials yields more significant decodability compared to the decoder based on no-pinged trials should be interpreted with caution because there are differences in N_{trials} between the two conditions that could partially or fully explain this effect. More generally, we found that the condition with more trials or more classes is by default more likely to yield significant p-values—but only if a true effect exist.

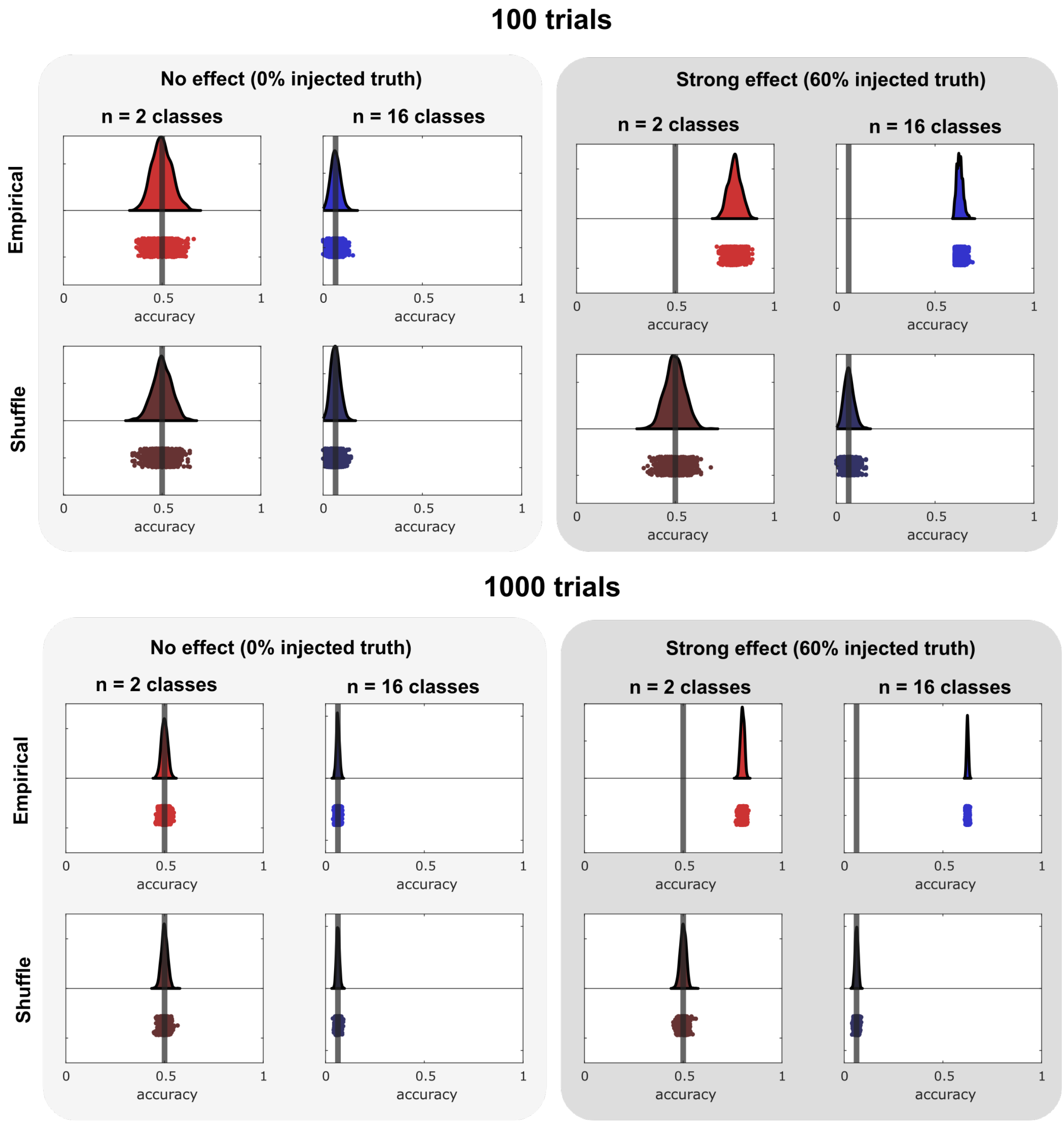

Supplementary Figure 7. The effects of class and trial number on decoding accuracy. Both the number of classes (columns) and trials (top vs. bottom half) influences the distance between shuffled and empirical distributions—but only if there is an effect in the data (left vs. right half).

These findings may be a manifestation of the classical notion of statistical power in statistical analysis but within the less intuitive context of decoding accuracy. Our interpretation then is not that N_{trials} and $N_{classes}$ must necessarily be equal between conditions for a statistical comparison to be meaningful. Rather, we wanted to err on the side of caution and ensure that analyses where power differences could possibly explain condition differences (e.g., Fig. 3 and Fig. 4A and 4B in the main text) do not

inform subsequent analyses and scientific interpretations by themselves. Instead, we supplemented each of the implicated analyses with additional rationale (in the case of Fig. 3) or analyses that do not involve empirical-to-shuffle decoding comparisons. Indeed, Fig. 4C and Fig. 4D involve direct comparisons between empirical and shuffled distributions, sidestepping the issue altogether.

4. Variance analysis

We evaluated how pings affect the variance of EEG data by comparing the variance across trials and channels between pinged and non-pinged trials during the retrieval phase of the experiment (Supplementary Figure 8). For the former analysis, we computed for each timepoint from 500 ms before retrieval cue to 2000 ms after retrieval cue how much the average activity across channels varies on a trial-by-trial basis (averaged across participants). For the latter, we calculated how much the average activity across trials varies between EEG channels (averaged across participants). Statistical analyses were carried out identically to the ping vs. no ping classifier analysis visualized in Figure 4C (see Methods for details), but with variance as the unit of comparison rather than decoding performance. We found no significant difference between the cross-trial or cross-channel variance for the two conditions (Wilcoxon signed-rank test; $p > 0.99$). We discuss the interpretation of these findings in the main body's discussion section.

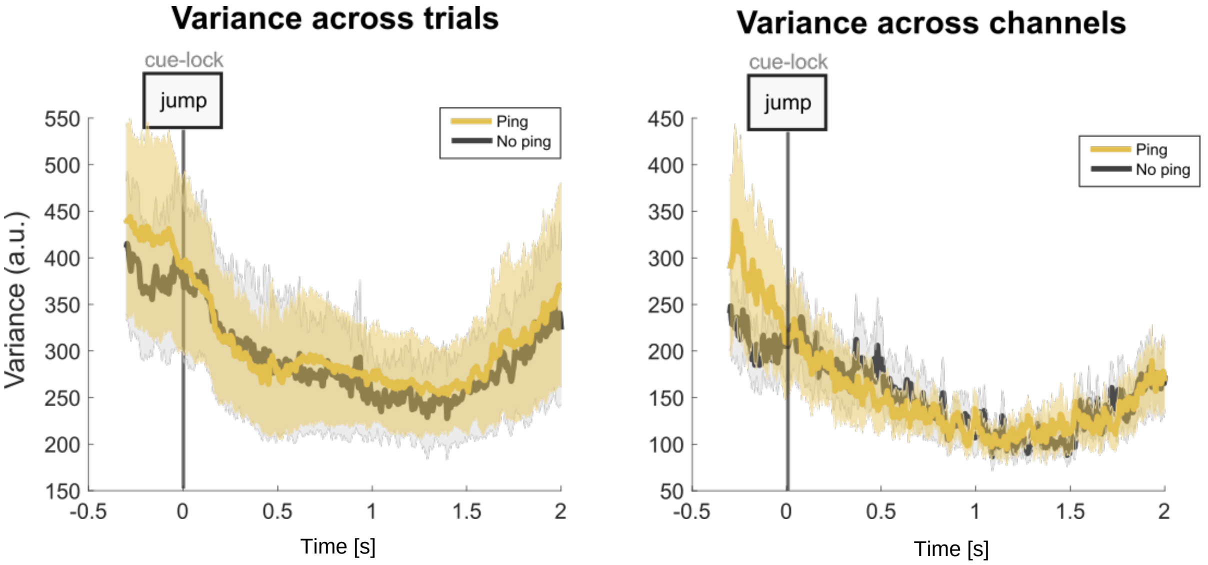

Supplementary Figure 8. Variance comparison between pinged and no-pinged trials during the retrieval phase of the experiment. We observe no significant difference in the average variance between conditions.
